# Associations of Obesity Measures with Subtypes of Ischemic Stroke in the ARIC Study

**DOI:** 10.2188/jea.JE20090186

**Published:** 2010-09-05

**Authors:** Hiroshi Yatsuya, Kazumasa Yamagishi, Kari E. North, Frederick L. Brancati, June Stevens, Aaron R. Folsom

**Affiliations:** 1 Division of Epidemiology and Community Health, School of Public Health, University of Minnesota, Minneapolis, MN, USA; 2 Department of Public Health, Graduate School of Medicine, Nagoya University, Nagoya, Japan; 3 Department of Public Health Medicine, Graduate School of Comprehensive Human Sciences, and Institute of Community Medicine, University of Tsukuba, Tsukuba, Japan; 4 Department of Epidemiology and Carolina Center for Genome Sciences, Gillings School of Global Public Health, University of North Carolina, Chapel Hill, NC, USA; 5 Departments of Medicine and Epidemiology, Johns Hopkins University, Baltimore, MD, USA; 6 Department of Nutrition, Gillings School of Global Public Health, University of North Carolina, Chapel Hill, NC, USA

**Keywords:** obesity, stroke, lacunar infarction, incidence, risk factors

## Abstract

**Background:**

Associations between obesity and lacunar, nonlacunar thrombotic, and cardioembolic stroke are not firmly established.

**Methods:**

Body mass index (BMI), waist circumference, and waist-to-hip ratio (WHR) were recorded at baseline between 1987 and 1989 in the Atherosclerosis Risk in Communities (ARIC) Study for 13 549 black and white adults who were aged from 45 to 64 years and had no history of cardiovascular disease or cancer. The incidence of ischemic stroke subtypes was ascertained from surveillance of hospital records over a median follow-up of 16.9 years. Cox proportional hazards regression analyses adjusted for age, sex, race, education, smoking status and cigarette years, usual ethanol intake, and leisure time sports index were used to estimate hazard ratios (HRs).

**Results:**

The ARIC sample at baseline was 43.8% men and 27.3% blacks; mean age was 53.9 years. Mean BMI, waist circumference, and WHR were 27.7 kg/m^2^, 96.8 cm, and 0.92, respectively. The associations of lacunar (*n* = 138), nonlacunar (*n* = 338), and cardioembolic (*n* = 122) ischemic stroke incidence with obesity measures were all generally positive and linear. The HRs for the highest versus lowest quintile of the 3 obesity measures ranged from 1.43–2.21 for lacunar stroke, 1.90–2.16 for nonlacunar stroke, and 2.37–2.91 for cardioembolic stroke.

**Conclusions:**

Although different pathophysiological mechanisms may exist, the incidences of lacunar, nonlacunar, and cardioembolic stroke were all significantly positively associated with the degree of obesity, regardless of the measure used.

## INTRODUCTION

Ischemic stroke consists of 3 major subtypes with different etiologies: lacunar, nonlacunar thrombotic, and cardioembolic. The risk factors for these subtypes may differ because of variation in the underlying vascular and non-vascular pathophysiologies of each subtype, such as lipohyalinosis and microatheroma, or atherosclerosis. For example, serum cholesterol level, atherosclerosis score, and the extent of artery stenosis were different in infarctions in penetrating artery regions (which correspond to lacunar stroke) and those in cortical artery regions (which presumably correspond to nonlacunar thrombotic stroke).^1^ Although overweight/obesity is generally considered a risk factor for ischemic stroke,^2^^–^^5^ differences in the associations of obesity with the subtypes of ischemic stroke have been less thoroughly studied and are not yet clear. A previous investigation in the ARIC Study found that body mass index and waist-to-hip ratio were positively associated with nonlacunar and cardioembolic ischemic stroke, but not with lacunar stroke, in a minimally adjusted model.^6^ However, some reports suggest a positive association between the degree of obesity and lacunar stroke incidence in women^7^ and with the prevalence of silent lacunar infarct identified by MRI.^8^ Thus, further studies on this topic are warranted, especially in an era in which obesity is increasing in most populations.

Both BMI and waist circumference are used in research and clinical settings for defining the health risks of obesity. In the present study, we described incidence rates and hazard ratios of ischemic stroke subtypes in relation to BMI, waist circumference, and WHR. We tested the hypotheses that there are differences in the associations according to subtype, and that any associations of obesity measures with ischemic stroke subtypes would be mediated by known stroke predictors (particularly hypertension and diabetes).

## METHODS

### Study population

The ARIC Study included a cohort of 15 792 persons between 45 and 64 years of age at recruitment in 1987–1989.^9^ Population samples were selected by probability sampling methods from Forsyth County, NC (*n* = 4035); Jackson, MS (blacks only, *n* = 3728); northwest suburbs of Minneapolis, MN (*n* = 4009); and Washington County, MD (*n* = 4020). Baseline response rates ranged from 46% in Jackson to 65% to 67% in the other 3 communities. Participants were subsequently contacted annually by telephone and at 3 additional clinic visits. The retention rate was 93% through 2005, and the rate did not differ appreciably between races.

### Baseline assessment

Body mass index (BMI: kg/m^2^) was calculated from measurements of weight to the nearest pound and height to the nearest centimeter, with the participants wearing a scrub suit and no shoes. The ratio of waist (umbilical level) to hip (maximum buttocks) circumference (waist-to-hip ratio: WHR) was calculated as a measure of fat distribution, in addition to waist circumference alone. The inter-technician reliability coefficients for waist and hip circumference and WHR were all *r* > 0.94.^10^

Questionnaires were used to assess educational level, cigarette smoking, alcohol drinking, leisure time sports index, use of antihypertensive and diabetic medications, and histories of physician-diagnosed diabetes, cancer, CHD, and stroke. The sports index was derived from questionnaire items on hours per week spent in up to 4 sports and the months per year each sport was played. By assuming a sport intensity level (light, moderate, or heavy), a sport score was calculated ranging from 1 (lowest) to 5 (highest).^11^ Prevalent CHD at baseline was defined for exclusion as a reported history of a physician-diagnosed myocardial infarction, prior myocardial infarction detected by electrocardiography, or prior cardiovascular surgery or coronary angioplasty.

Three sitting brachial blood pressure measurements were taken with a random-zero sphygmomanometer on the right arm after 5 minutes’ rest; the last 2 measurements were averaged. The manual for ARIC blood pressure measurement can be accessed online (http://www.cscc.unc.edu/aric/). Blood was drawn after an 8-hour fasting period, with minimal trauma, from an antecubital vein. The proportion of subjects that met the 8-hour fasting criterion was 97%. Blood levels of von Willebrand factor (vWF), glucose, high-density lipoprotein (HDL) cholesterol, and albumin were measured centrally by standard methods. Prevalent diabetes was defined a history of or treatment for diabetes, a fasting glucose level ≥126 mg/dl, or a casual blood glucose level ≥200 mg/dl.

### Ascertainment of incident stroke

Ischemic strokes that occurred by December 31, 2005 (median follow-up 16.9 years) were included in the present study. During annual telephone contacts, interviewers asked each ARIC participant to list all hospitalizations during the past year; hospital records were obtained. In addition, all local hospitals annually provided lists of stroke discharges (International Classification of Diseases, Ninth Revision, Clinical Modification codes 430–438), which were scrutinized for discharges of ARIC participants. Details on quality assurance for ascertainment and classification of stroke are described elsewhere.^12^ Briefly, the stroke diagnosis was assigned according to criteria adapted from the National Survey of Stroke.^13^ Strokes secondary to trauma, neoplasm, hematological abnormality, infection, or vasculitis were excluded, and a focal deficit lasting <24 hours was not considered a stroke. Out-of-hospital stroke was not ascertained or validated; thus, these potential stroke events were not included. A stroke was classified as ischemic when a brain CT or MRI revealed acute infarction or showed no evidence of hemorrhage. All definite ischemic strokes were further classified as lacunar, nonlacunar thrombotic, or cardioembolic on the basis of recorded neuroimaging results. A stroke was classified as lacunar when 2 criteria were met: (1) typical location of the infarct (basal ganglia, brain stem, thalamus, internal capsule, or cerebral white matter) and (2) infarct size of ≤2 cm or unstated size.^14^ Definite or probable cardioembolic stroke required either (1) autopsy evidence of an infarcted area in the brain and a source of possible cerebral emboli in a vessel or the presence of an embolus in the brain, or, (2) recorded medical evidence of a possible non-carotid source of embolus such as moderate or more severe valvular heart disease, atrial fibrillation, cardiac or arterial procedure (eg, cardiac catheterization, open heart surgery, cerebral angiography, and carotid endarterectomy), or intracardiac thrombus. Definite or probable ischemic strokes that were not deemed lacunar or cardioembolic—including atherothrombotic and unclassified thrombotic strokes—were labeled nonlacunar. Along with computer-based classification, cases were independently reviewed by a physician who was provided with a detailed report of the information abstracted from the medical record, as well as the full discharge summary, the CT and MRI scan reports, reports from any neurological consults, and admission history. The final diagnosis was determined by agreement of the computer and reviewer classification. On the rare occasion when there was a disagreement between computer and reviewer classifications, the diagnosis was adjudicated by a second physician-reviewer. CT or MRI was available for all ischemic stroke cases except 1 cardioembolic stroke that was classified using carotid artery ultrasound and clinical information. The 92 hemorrhagic strokes identified by ARIC were censored at the time of their occurrence.

### Statistical analysis

Of the 15 744 blacks and whites in ARIC, we excluded 1787 participants (blacks: 365, whites: 1422) who, at baseline, had a prevalent stroke, CHD, or cancer, because CVD treatment and associated behavioral change or cancer-induced weight loss could confound the association between obesity measures and stroke. Participants lacking baseline measurements of BMI or waist or hip circumference (*n* = 32) were also excluded. Those with missing values for potential confounding variables, including leisure time sport index, smoking status and cigarette-years of smoking, usual ethanol intake, or educational level, were also excluded (*n* = 376), leaving a final sample of 5930 men and 7619 women (*n* = 13 549 in total). Sensitivity analyses were also performed after truncating the sample at the 1st and 99th percentile of each obesity measure in an attempt to exclude any impact of extreme values on the associations.

Since no interactions of race or sex with obesity measure quintiles was observed (*P* > 0.1, Wald test with 12 degrees of freedom), analyses were done with the race- and sex-groups combined. Age-, sex-, and race-adjusted means and proportions among ischemic stroke subtypes were calculated by a general linear model and tested with Tukey-Kramer adjustment. Cox proportional hazards regression was used to calculate age-, sex-, and race-adjusted, and multivariate-adjusted, hazard ratios (HRs) and their 95% confidence intervals (CIs) for subtypes of ischemic stroke incidence in relation to quintiles of the obesity measures, with the first quintile as the reference category. Quintile cutoff values of each obesity measure were created by averaging the 4 race-sex–specific quintile cutoff values. They were 23.9, 26.2, 28.6, and 32.0 (kg/m^2^) for BMI; 86, 92, 99, and 107 (cm) for waist circumference; and 0.87, 0.91, 0.94, and 0.98 for WHR. The first model (model I) adjusted for age, sex, race, smoking status (current, past, or never), cigarette-years of smoking, usual ethanol intake (grams/week), educational level (high school graduate or not), and leisure time sport index score (1.0–1.9, 2.0–2.4, 2.5–2.9, 3.0–5.0). In a mediation model (Model II, additional subjects excluded, *n* = 288), we further adjusted simultaneously for systolic blood pressure, use of antihypertensive medication, prevalent diabetes, and blood levels of HDL cholesterol, vWF, and albumin in light of a previous ARIC report that identified these factors as predictors of incident stroke.^15^ A trend test was performed by assigning the median value of each quintile to corresponding individuals and treating it as a continuous variable in the model. When there was an indication of a nonlinear association on quintile categorical analysis, statistical significance of the quadratic term for the obesity measure (continuous) was evaluated. Additionally, for each ischemic stroke subtype, we performed analysis stratified by hypertension, which was defined as either systolic/diastolic blood pressure ≥140/90 mm Hg or current use of medication for hypertension. Interactions of hypertension with obesity measure quintile were tested in Model I using a Wald test with 4 degrees of freedom and the criterion probability level (p) set at 0.1. The assumption of hazard proportionality was tested by a model including follow-up time by obesity measure quintile interaction. The follow-up time was first examined using a continuous scale, and then dichotomized at year 10. All statistical analyses were performed using SAS 9.2.

## RESULTS

Waist circumference quintile was positively associated with age, while the proportions of men and blacks were lowest in the first quintile (Table 1). Although the prevalence of current smoking was inversely associated with waist circumference quintile, cigarette-years of smoking among ever smokers was positively associated with waist circumference quintile. The proportion of subjects with more than a high school education was higher in lower waist circumference quintiles.

**Table 1.** Age-, sex, and race-adjusted baseline characteristics of participants according to waist circumference quintile, ARIC 1988–1990

Characteristics		Waist circumference quintile					*P*
	
		Q1	Q2	Q3	Q4	Q5	
	Range in cm (median)	52–86 (81)	87–92 (90)	93–99 (96)	100–107 (103)	108–178 (115)	
Age (y)^a^		52.9	53.6	54.1	54.3	54.5	<0.0001
Men (%)^b^		20.8	44.7	56.1	57.4	42.0	<0.0001
Blacks (%)^c^		23.7	27.0	25.1	27.1	34.3	<0.0001
Body mass index (kg/m^2^)		22.5	25.1	26.8	29.4	35.2	<0.0001
Waist circumference (cm)		79.6	89.6	95.9	103.2	117.5	<0.0001
Hip circumference (cm)		95.9	99.9	102.8	107.1	118.4	<0.0001
Waist-to-hip ratio		0.83	0.90	0.94	0.97	0.99	<0.0001
More than high school diploma (%)		49.0	46.2	47.2	43.5	36.9	<0.0001
Current smoker (%)		31.7	27.4	24.3	23.1	20.0	<0.0001
Cigarette-years (among ever smokers)		487	491	528	573	576	<0.0001
Usual ethanol intake (g/week)		39.0	43.6	47.1	47.2	35.7	<0.0001
Leisure time sports index (≥3) (%)		30.4	31.2	31.3	26.1	19.7	<0.0001
Antihypertensive medication (%)		17.6	23.0	25.4	31.4	43.7	<0.0001
Diabetes mellitus (%)		3.2	6.0	8.5	14.0	23.2	<0.0001
Systolic blood pressure (mm Hg)		114.8	119.4	121.3	122.7	128.1	<0.0001
Diastolic blood pressure (mm Hg)		70.3	73.3	74.2	74.8	77.0	<0.0001
High-density lipoprotein cholesterol (mg/dl)		63.3	53.9	49.8	46.5	45.8	<0.0001
von Willebrand factor (%)		111.1	112.7	114.5	117.1	130.1	<0.0001
Albumin (g/dl)		3.9	3.9	3.9	3.9	3.8	<0.0001

^a^Adjusted for sex and race.

^b^Adjusted for age and race.

^c^Adjusted for age and sex.

During a median of 16.9 years of follow-up (max = 19.1 years), there were 598 incident ischemic strokes, 138 of which were lacunar, 338 nonlacunar, and 122 cardioembolic. At baseline, mean BMI, waist circumference, and WHR were significantly higher in those who developed nonlacunar or cardioembolic stroke, as compared with subjects who did not develop ischemic stroke. Although WHR was significantly higher in those who developed lacunar stroke than in those without an ischemic stroke, BMI and waist circumference were not significantly different. Blacks were more likely than whites to develop ischemic stroke, especially lacunar stroke. The crude incidence rate of lacunar stroke in blacks was more than 4-fold that of whites (1.51 versus 0.34 per 1000 person-years, data not shown in table). The proportion of subjects taking antihypertensive medications and mean systolic blood pressure were similarly higher in those who developed any ischemic stroke subtype, as compared with those free of ischemic stroke. The prevalence of diabetes mellitus was highest in lacunar stroke cases, followed by cardioembolic and nonlacunar ischemic stroke cases.

As shown in Table 2, the HRs of lacunar stroke were, in general, positively and linearly associated with waist circumference and WHR quintiles, but less so with BMI. The HR of lacunar stroke for the highest quintile of BMI was only 1.43 (95% CI: 0.84–2.45), and continuous BMI showed no significant association (HR1 per standard deviation: 1.15, 95% CI: 0.98–1.34). However, the inclusion of a quadratic BMI (continuous) variable also did not support departure from linearity (*P* for quadratic BMI = 0.29). Adjustment for potential mediating factors (model II)—particularly diabetes, hypertension, and HDL cholesterol—significantly attenuated the associations of most obesity measures with lacunar stroke.

**Table 2.** Incidence and hazard ratios (95% CI) of lacunar ischemic stroke according to obesity measure quintile or per standard deviation, ARIC, 1987–2005

	Quintile					Trend *P*	HR1
	
	Q1	Q2	Q3	Q4	Q5		
Body mass index (kg/m^2^)							
Range (median)	14.4– <23.9 (22.2)	23.9– <26.2 (25.1)	26.2– <28.6 (27.3)	28.6– <32.0 (30.1)	32.0– <65.9 (35.1)		
Number of cases	25	16	20	42	35		
Incidence rate	0.5	0.4	0.5	1.1	0.9		
Age-, sex-, and race-adjusted	1 (Reference)	0.65 (0.34–1.21)	0.75 (0.41–1.35)	1.59 (0.97–2.62)	1.28 (0.75–2.16)	0.034	1.12 (0.95–1.31)
Model I	1 (Reference)	0.71 (0.38–1.32)	0.81 (0.45–1.47)	1.78 (1.07–2.95)	1.43 (0.84–2.45)	0.015	1.15 (0.98–1.34)
Model II	1 (Reference)	0.55 (0.29–1.06)	0.52 (0.28–0.98)	1.10 (0.64–1.88)	0.54 (0.30–0.98)	0.29	0.84 (0.69–1.01)
Waist circumference (cm)							
Range (median)	52–86 (81)	87–92 (90)	93–99 (96)	100–107 (103)	108–178 (115)		
Number of cases	21	16	22	35	44		
Incidence rate	0.4	0.5	0.5	0.8	1.1		
Age-, sex-, and race-adjusted	1 (Reference)	0.92 (0.48–1.77)	0.90 (0.49–1.64)	1.64 (0.95–2.83)	1.98 (1.18–3.34)	0.0006	1.22 (1.05–1.43)
Model I	1 (Reference)	0.97 (0.50–1.86)	0.95 (0.52–1.74)	1.73 (1.00–2.99)	2.10 (1.24–3.56)	0.0004	1.24 (1.06–1.45)
Model II	1 (Reference)	0.70 (0.35–1.40)	0.61 (0.33–1.14)	0.90 (0.50–1.63)	0.78 (0.43–1.40)	0.80	0.88 (0.73–1.06)
Waist-to-hip ratio							
Range (median)	0.49– <0.87 (0.82)	0.87– <0.91 (0.89)	0.91– <0.94 (0.93)	0.94– <0.98 (0.96)	0.99– <1.39 (1.01)		
Number of cases	19	16	25	38	40		
Incidence rate	0.4	0.5	0.7	0.8	0.9		
Age-, sex-, and race-adjusted	1 (Reference)	1.14 (0.58–2.23)	1.58 (0.85–2.92)	2.10 (1.18–3.74)	2.40 (1.35–4.27)	0.0005	1.41 (1.16–1.71)
Model I	1 (Reference)	1.12 (0.57–2.20)	1.52 (0.82–2.82)	2.02 (1.14–3.60)	2.21 (1.24–3.95)	0.0015	1.37 (1.12–1.66)
Model II	1 (Reference)	0.77 (0.38–1.55)	0.98 (0.52–1.82)	0.98 (0.53–1.79)	0.87 (0.47–1.61)	0.80	0.94 (0.76–1.17)

HR, hazard ratio; CI, confidence interval.

Model I: Adjusted for age, sex, race, education, smoking status, cigarette years, usual ethanol intake, and leisure time sports index.

Model II: Model I + systolic blood pressure, antihypertensive medication, diabetes, and blood levels of high-density lipoprotein cholesterol, von Willebrand factor, and albumin.

Trend test was performed by assigning the median value of each quintile to corresponding individuals and treating it as a continuous variable in the model.

HR1: HR per 1 standard deviation (5.4 kg/m^2^, 13.9 cm, or 0.078) increment of body mass index, waist circumference, or waist-to-hip ratio, respectively.

Nonlacunar ischemic stroke was positively and strongly associated with all obesity measures in quintiles, and on a continuous scale (Table 3). The association of nonlacunar stroke incidence with obesity measures was attenuated with adjustment for mediating factors, but incompletely so for WHR (HR1: 1.16, 95% CI: 1.01–1.34 in Model II). The results of analyses for cardioembolic stroke were similar to those for nonlacunar stroke (Table 4). The HRs of cardioembolic stroke for the highest quintiles of obesity measures ranged from 2.37 through 2.91.

**Table 3.** Incidence and hazard ratios (95% CI) of nonlacunar ischemic stroke according to obesity measure quintile or per standard deviation, ARIC, 1987–2005

	Quintile					Trend *P*	HR1
	
	Q1	Q2	Q3	Q4	Q5		
Body mass index (kg/m^2^)							
Range (median)	14.4– <23.9 (22.2)	23.9– <26.2 (25.1)	26.2– <28.6 (27.3)	28.6– <32.0 (30.1)	32.0– <65.9 (35.1)		
Number of cases	59	57	74	63	85		
Incidence rate	1.2	1.3	1.7	1.6	2.3		
Age-, sex-, and race-adjusted	1 (Reference)	1.02 (0.71–1.47)	1.27 (0.90–1.80)	1.19 (0.83–1.71)	1.73 (1.23–2.43)	0.0006	1.19 (1.07–1.32)
Model I	1 (Reference)	1.10 (0.76–1.58)	1.36 (0.96–1.93)	1.30 (0.91–1.87)	1.90 (1.34–2.68)	0.0001	1.22 (1.10–1.35)
Model II	1 (Reference)	1.04 (0.71–1.52)	1.22 (0.85–1.76)	1.02 (0.69–1.50)	1.14 (0.77–1.68)	0.63	1.00 (0.89–1.12)
Waist circumference (cm)							
Range (median)	52–86 (81)	87–92 (90)	93–99 (96)	100–107 (103)	108–178 (115)		
Number of cases	48	49	70	66	105		
Incidence rate	1.0	1.4	1.4	1.6	2.6		
Age-, sex-, and race-adjusted	1 (Reference)	1.23 (0.83–1.84)	1.18 (0.82–1.72)	1.30 (0.89–1.90)	2.08 (1.47–2.93)	<0.0001	1.28 (1.15–1.41)
Model I	1 (Reference)	1.28 (0.85–1.91)	1.25 (0.86–1.82)	1.36 (0.93–1.99)	2.16 (1.52–3.06)	<0.0001	1.29 (1.16–1.43)
Model II	1 (Reference)	1.22 (0.80–1.85)	1.08 (0.73–1.60)	1.05 (0.70–1.58)	1.31 (0.88–1.95)	0.23	1.05 (0.93–1.18)
Waist-to-hip ratio							
Range (median)	0.49– <0.87 (0.82)	0.87– <0.91 (0.89)	0.91– <0.94 (0.93)	0.94– <0.98 (0.96)	0.99– <1.39 (1.01)		
Number of cases	45	43	56	73	121		
Incidence rate	0.9	1.3	1.5	1.6	2.7		
Age-, sex-, and race-adjusted	1 (Reference)	1.25 (0.82–1.90)	1.33 (0.89–1.99)	1.40 (0.95–2.07)	2.28 (1.58–3.29)	<0.0001	1.43 (1.26–1.62)
Model I	1 (Reference)	1.21 (0.79–1.85)	1.30 (0.87–1.95)	1.38 (0.93–2.04)	2.13 (1.48–3.09)	<0.0001	1.40 (1.23–1.59)
Model II	1 (Reference)	1.12 (0.72–1.73)	1.09 (0.71–1.66)	1.01 (0.67–1.53)	1.34 (0.89–2.01)	0.16	1.16 (1.01–1.34)

HR, hazard ratio; CI, confidence interval.

Model I: Adjusted for age, sex, race, education, smoking status, cigarette years, usual ethanol intake, and leisure time sports index.

Model II: Model I + systolic blood pressure, antihypertensive medication, diabetes, and blood levels of high-density lipoprotein cholesterol, von Willebrand factor, and albumin.

Trend test was performed by assigning the median value of each quintile to corresponding individuals and treating it as a continuous variable in the model.

HR1: HR per 1 standard deviation (5.4 kg/m^2^, 13.9 cm, or 0.078) increment of body mass index, waist circumference, or waist-to-hip ratio, respectively.

**Table 4.** Incidence and hazard ratios (95% CI) of cardioembolic stroke according to obesity measure quintile or per standard deviation, ARIC, 1987–2005

	Quintile					Trend *P*	HR1
	
	Q1	Q2	Q3	Q4	Q5		
Body mass index (kg/m^2^)							
Range (median)	14.4– <23.9 (22.2)	23.9– <26.2 (25.1)	26.2– <28.6 (27.3)	28.6– <32.0 (30.1)	32.0– <65.9 (35.1)		
Number of cases	15	19	24	30	34		
Incidence rate	0.3	0.4	0.6	0.8	0.9		
Age-, sex-, and race-adjusted	1 (Reference)	1.31 (0.66–2.58)	1.58 (0.82–3.03)	2.18 (1.16–4.06)	2.67 (1.44–4.95)	0.0003	1.39 (1.19–1.62)
Model I	1 (Reference)	1.36 (0.69–2.69)	1.66 (0.86–3.19)	2.34 (1.25–4.40)	2.91 (1.55–5.45)	0.0001	1.42 (1.21–1.67)
Model II	1 (Reference)	1.13 (0.57–2.25)	1.23 (0.63–2.38)	1.49 (0.77–2.86)	1.28 (0.66–2.50)	0.44	1.08 (0.90–1.29)
Waist circumference (cm)							
Range (median)	52–86 (81)	87–92 (90)	93–99 (96)	100–107 (103)	108–178 (115)		
Number of cases	15	14	25	33	35		
Incidence rate	0.3	0.4	0.5	0.8	0.9		
Age-, sex-, and race-adjusted	1 (Reference)	1.13 (0.54–2.35)	1.37 (0.72–2.63)	2.11 (1.14–3.93)	2.27 (1.23–4.18)	0.0009	1.42 (1.21–1.68)
Model I	1 (Reference)	1.13 (0.54–2.36)	1.41 (0.73–2.70)	2.20 (1.18–4.10)	2.37 (1.28–4.39)	0.0006	1.45 (1.23–1.72)
Model II	1 (Reference)	0.95 (0.45–1.98)	0.99 (0.51–1.94)	1.33 (0.70–2.56)	1.03 (0.53–1.98)	0.77	1.07 (0.89–1.29)
Waist-to-hip ratio							
Range (median)	0.49– <0.87 (0.82)	0.87– <0.91 (0.89)	0.91– <0.94 (0.93)	0.94– <0.98 (0.96)	0.99– <1.39 (1.01)		
Number of cases	15	13	20	33	41		
Incidence rate	0.3	0.4	0.5	0.7	0.9		
Age-, sex-, and race-adjusted	1 (Reference)	1.16 (0.55–2.46)	1.49 (0.75–2.98)	2.02 (1.06–3.83)	2.58 (1.37–4.85)	0.0006	1.45 (1.18–1.79)
Model I	1 (Reference)	1.15 (0.54–2.44)	1.47 (0.74–2.94)	2.01 (1.06–3.83)	2.54 (1.35–4.80)	0.0007	1.44 (1.17–1.78)
Model II	1 (Reference)	0.96 (0.45–2.03)	1.08 (0.54–2.16)	1.24 (0.64–2.40)	1.17 (0.60–2.29)	0.52	1.06 (0.84–1.33)

HR, hazard ratio; CI, confidence interval.

Model I: Adjusted for age, race, sex, education, smoking status, cigarette years, usual ethanol intake, and leisure time sports index.

Model II: Model I + systolic blood pressure, antihypertensive medication, diabetes, and blood levels of high-density lipoprotein cholesterol, von Willebrand factor, and albumin.

Trend test was performed by assigning the median value of each quintile to corresponding individuals and treating it as a continuous variable in the model.

HR1: HR per 1 standard deviation (5.4 kg/m^2^, 13.9 cm, or 0.078) increment of body mass index, waist circumference, or waist-to-hip ratio, respectively.

Sensitivity analyses excluding subjects with extreme values of obesity measures produced similar findings, except for the association between BMI and lacunar stroke incidence, where the HR per 5.4 kg/m^2^ increment (corresponding to HR1 of Model I in Table 2) became statistically significant (HR: 1.29, *P* = 0.006).

Hypertension modified the associations of obesity measure quintiles with lacunar stroke (Table 5, *P* for interaction <0.05), but no such effect modification was observed for nonlacunar or cardioembolic stroke (*P* for interaction >0.3). Significant positive associations between obesity measures and incidence of lacunar stroke were present only in subjects without hypertension. In contrast, obesity was not associated with the incidence of lacunar stroke among hypertensives. Subjects with hypertension had a significantly higher BMI than those without hypertension (29.5 versus 26.7 kg/m^2^).

**Table 5.** Incidence and hazard ratios (95% CI) of lacunar stroke according to obesity measure quintile in participants with or without baseline hypertension, ARIC, 1987–2005

	Quintile^a^					Trend *P*
	
	Q1	Q2	Q3	Q4	Q5	
Without hypertension (46 cases)						
Body mass index						
Incidence rate	0.2	0.1	0.2	0.9	0.4	
No. of cases/person years	10/40 191	4/31 002	5/27 688	21/22 799	6/16 072	
Model I	1 (Reference)	0.48 (0.15–1.54)	0.64 (0.22–1.89)	3.54 (1.64–7.63)	1.75 (0.62–4.94)	0.0042
Waist circumference						
Incidence rate	0.2	0.2	0.2	0.6	0.7	
No. of cases (incidence rate)	7/37 839	4/24 098	6/32 443	15/24 680	14/18 693	
Model I	1 (Reference)	0.75 (0.22–2.59)	0.85 (0.28–2.57)	2.72 (1.08–6.86)	3.71 (1.47–9.36)	<0.001
Waist-to-hip ratio						
Incidence rate	0.2	0.1	0.2	0.4	0.8	
No. of cases (incidence rate)	7/37 266	2/22 616	6/25 203	12/28 449	19/24 217	
Model I	1 (Reference)	0.37 (0.08–1.83)	0.94 (0.30–2.94)	1.71 (0.62–4.70)	3.12 (1.19–8.19)	0.0018

With hypertension (92 cases)						
Body mass index						
Incidence rate	1.4	1.0	1.0	1.3	1.3	
No. of cases (incidence rate)	15/11 022	12/12 595	15/14 871	21/16 006	29/21 559	
Model I	1 (Reference)	0.66 (0.31–1.41)	0.64 (0.31–1.32)	0.79 (0.40–1.55)	0.76 (0.40–1.47)	0.80
Waist circumference						
Incidence rate	1.3	1.1	1.0	1.2	1.4	
No. of cases (incidence rate)	14/11 181	12/10 555	16/15 863	20/16 545	30/21 907	
Model I	1 (Reference)	0.84 (0.39–1.83)	0.74 (0.36–1.53)	0.92 (0.46–1.84)	1.04 (0.55–1.98)	0.61
Waist-to-hip ratio						
Incidence rate	0.9	1.3	1.5	1.4	1.0	
No. of cases (incidence rate)	12/13 221	14/10 466	19/13 057	26/18 471	21/20 837	
Model I	1 (Reference)	1.37 (0.63–3.00)	1.51 (0.72–3.19)	1.59 (0.78–3.24)	1.25 (0.59–2.64)	0.56

CI, confidence interval.

Model I: Adjusted for age, sex, race, education, smoking status, cigarette years, usual ethanol intake, and leisure time sports index.

Trend test was performed by assigning the median value of each quintile to corresponding individuals and treating it as a continuous variable in the model.

*P* values for the interactions for quintiles of body mass index, waist circumference, and waist-to-hip ratio by hypertension were 0.024, 0.060, and 0.006, respectively.

^a^Cut-off values for obesity measure quintiles are the same as in Tables 2, 3, and 4.

## DISCUSSION

In this analysis of data from the ARIC cohort, all obesity measures were positively and similarly associated with all ischemic stroke subtypes. These associations were slightly stronger than in a previous ARIC report that had fewer events and did not examine waist circumference.^6^

The present findings are consistent in part with the Hisayama Study, which found a positive association between BMI and lacunar stroke incidence in Japanese women.^7^ However, there were no BMI associations with any ischemic stroke subtype in men or with atherothrombotic or cardioembolic stroke in women. The large difference in the degree of obesity between the ARIC and Hisayama studies precludes direct comparisons (mean BMIs in the Hisayama Study were 21.5 kg/m^2^ in men and 21.7 kg/m^2^ in women, compared with 27.7 kg/m^2^ for both sexes in the ARIC Study). The ARIC finding may also be consistent with a cross-sectional study that found a positive association between abdominal obesity and the prevalence of silent lacunar infarct identified by MRI in apparently healthy Japanese men and women aged 40 to 59 years.^8^ Another study^16^ found that a BMI ≥27 kg/m^2^ was not associated with any ischemic stroke subtype after adjusting for age, sex, social class, hypertension, and hypercholesterolemia. Since mediating variables were included, these results are consistent with our findings from the mediation analyses. Nevertheless, further investigations in populations with various degrees of obesity, and different ethnicities and sexes, are essential to confirm the present findings.

Mediating factors together explained all the association of obesity measures with each ischemic stroke subtype, but no single mediating factor wholly accounted for the associations, except in the case of lacunar stroke analyses, in which hypertension, diabetes mellitus, and HDL cholesterol each attenuated the significant association. Coefficients for waist circumference (HR1) changed 44% after adding systolic blood pressure and antihypertensive medication to Model I. The extent of the change was 72% in the case of diabetes mellitus and 35% for HDL cholesterol. Statistically significant associations of waist circumference with lacunar stroke incidence disappeared in all cases. Each of these variables explained some associations between waist circumference and nonlacunar thrombotic or cardioembolic stroke (changes in the coefficients from Model I ranged from 12% to 38%), but the statistical significance of waist circumference remained. It was only when all these variables were entered simultaneously that the significant association was attenuated (*P* = 0.13 for both nonlacunar thrombotic and cardioembolic stroke). Given the strong and probably causal association between obesity and hypertension, diabetes mellitus, and HDL cholesterol, obesity would be an important target for the prevention of any subtype of ischemic stroke.

We found no associations between obesity measures and lacunar stroke incidence in subjects with hypertension. This finding appears to be consistent with a prospective study of hypertensive adults aged 60 years or older that found no associations between BMI and any ischemic stroke subtype.^17^ We performed several additional analyses in an attempt to find a possible mechanism related to the null association; however, neither additional adjustment for potential mediating variables (excluding subjects with less than 5 years of follow-up) nor limiting analyses to either race or sex changed the null association in hypertensives. Other established risk factors, such as elevated systolic blood pressure, diabetes mellitus, and low HDL cholesterol, were all positively associated with lacunar stroke incidence in subjects with hypertension. Although the underlying mechanism remains unknown, it may have been due to a high incidence rate of lacunar stroke in hypertensives in the lowest BMI and waist circumference quintiles. Other dietary or lifestyle factors may have existed in these individuals that predisposed them to lacunar stroke over the effect of obesity.^18^ On the other hand, confidence intervals were wide and it is possible that the null finding was a chance result.

There are several limitations of this study that warrant discussion. Although neuroimaging reports and clinical features were used to classify ischemic stroke cases into subtypes, some cases may have been misclassified. Subtypes were classified via a review of medical records and neuroimaging reports, rather than by direct examination of patients or images. The medical record classification process did not use the obesity status or other risk factors of stroke cases, but reviewers were not blinded to such information. This might have lead to some potential biases in the associations. Further, since we used a maximum diameter of 20 mm in our study, there is the possibility of misclassification of nonlacunar thrombotic stroke into lacunar stroke; however, we minimized this possibility by prioritizing neuroimaging reports that explicitly stated that the infarct was not lacunar, even if the image itself met the criterion. Any misclassification might have distorted the obesity and lacunar stroke association. Nevertheless, the size criterion for the lacunar infarct has also been debated^19^; future studies utilizing advanced imaging would be ideal. Although we did not find any effect modification by race or sex, the number of cases was limited, so we could not conduct sex- or race-specific analyses. The present sample consisted of US blacks and whites, most of which were from a single center, which limits the generalizability of our findings to other cultural or socio-economic contexts. Finally, we used regression rather than structural equation modeling to examine mediation.

A strength of the present study is that we analyzed the association of obesity measures and ischemic stroke subtypes using prospective population-based data, including both blacks and whites, with relatively large numbers of events. No prior prospective studies have specifically addressed in detail the association between obesity and ischemic stroke subtypes.

In conclusion, lacunar, nonlacunar, and cardioembolic stroke were all significantly positively associated with obesity measures. Although different pathophysiological mechanisms may exist relating obesity and each subtype, the prevention and control of obesity has the potential to reduce the burden of stroke in the United States. Further studies in other populations are needed.
